# Tribute to James Trussell (1949–2018)

**DOI:** 10.1186/s40834-019-0096-y

**Published:** 2019-08-09

**Authors:** Deborah Kowal

**Affiliations:** Contraceptive Technology, Decatur, GA USA

**Keywords:** James Trussell, Contraceptive efficacy, Contraceptive effectiveness, Emergency contraception, Medical abortion

## Main text

James Trussell, who passed away at age 69 on December 26, 2018, leaves behind invaluable contributions to our field. Professor Trussell was a resolute champion for the evidentiary science in reproductive health, an ingenious researcher contributing substantially to that scientific evidence, and a decided advocate for reproductive choice. He generously and eloquently shared his understanding of the science with the “depth of a scholar and the history of a sage” [1].

An internationally known researcher, Professor Trussell leaves big footprints in the footnotes of scientific literature, having published more than 400 scientific articles in peer-reviewed journals. It is fitting, therefore, that this tribute, too, include footnotes. His commitment to data, his intellectual honesty, and his compulsion for precision and accuracy prompted colleagues to call him “the methodologic conscience of family planning research” [2]. He voiced his concerns when claims were made beyond the existing evidence, even if those concerns rankled his peers or dissenters. However, he focused his scrutiny on his own work as well, even if it could introduce doubt about his own previous findings. As an example, he had the “intellectual honesty to consider that emergency contraceptives might have a post-ovulatory impact when the efficacy rates were higher than expected. It later turned out that there was another explanation for the data, but he … openly discussed the possibility in a journal article, even though it gave ammunition to those who opposed emergency contraception” [3].

While he was tough and uncompromising in regard to scientific rigor, he was often sentimentally loyal and tender-hearted in his relationships with friends and close colleagues. He encouraged and consulted widely with health practitioners in their clinical research. “His piercing intellect and commitment to reproductive health inspired generations of young people to work in this field. He was unfailingly interested in all aspects of reproductive and sexual health and a great encouragement to many of us as we started out in the field” [4].

It is not a surprise to learn that he precociously co-authored his first book on family planning upon graduation from college in 1971 [5]. His interest in the field had begun 4 years earlier, just after he finished high school, when he inspired Dr. Robert A. Hatcher to start a summer internship in family planning. The following year, he began as the first intern in that internship, which continued for 30 more years and introduced 600 students to family planning, many of whom went on to be influential in the field of women’s reproductive health [6]. In 1972, Professor Trussell and Dr. Hatcher co-authored *Women in Need: a far-sighted family planning program designed to halt the epidemic of unwanted babies* [7]. That same year, the future Professor Trussell first contributed to Dr. Hatcher’s well-known reference text *Contraceptive Technology*, becoming a co-author in 1984 and continuing through the current 21st edition (2018).

Professor Trussell leaves behind a tremendous body of work, serving the fields of reproductive health, demography, economics, and more. Most known for his research in the evolving methodologies for estimating and interpreting contraceptive efficacy, Professor Trussell published meta-analyses of contraceptive failure rates. He was first to distinguish between the failure rates associated with typical use of contraceptive methods and the rates associated with perfect use. His landmark summary table on contraceptive failure during the first year of contraceptive use [8] is the most cited data in the family planning literature. For many years this table has been included in all FDA-approved contraceptive package inserts, guiding women and their health care providers in making evidence-based clinical decisions about contraceptive selection.

In more than 60 publications, Trussell explored the efficacy and mechanism of action of emergency contraceptives and actively promoted making emergency contraception more widely available as an important step in helping women reduce their risk of unintended pregnancy. In 1994, he established an emergency contraceptive website (not-2-late.com) [9]—an evidence-based source of information for individual women and clinicians; nearly 800,000 unique viewers visit the site each month. His work was influential in getting emergency contraceptive pills approved for use by all U.S. women, including teenagers [10].

Professor Trussell co-authored a series of papers on the cost-effectiveness of contraception [11] that provided evidence supporting the U.S. Affordable Care Act mandate that health insurance cover contraceptive supplies and services with no deductible or copay. This evidence had previously provided evidence encouraging increased insurance coverage of contraceptive methods.

With colleagues from Planned Parenthood Federation of America, he published a series of papers that changed the delivery of medical abortion worldwide. The group found that the infection-caused mortality rate following medical abortion declined by 100% following a change from vaginal to buccal administration of misoprostol combined with screen-and-treat or, far more commonly, routine antibiotic coverage [12].

Professor Trussell lent a demographic eye to the dynamics of reproductive behavior and population change. Several editions of *Contraceptive Technology* featured his chapter on the topic. His method schedules for estimating basic demographic parameters from inaccurate and incomplete data [13] is especially valuable in developing countries with poor infrastructures for reporting vital statistics. He published methods for estimating mortality, age at first marriage, and natural fertility.

Although not a clinician, Professor Trussell participated in the development of the CDC U.S. Medical Eligibility Criteria (as well as WHO’s Medical Eligibility Criteria) and the Selected Practice Recommendations for Contraceptive Use in the United States. He was on the National Medical Committee for the Planned Parenthood Federation of America, a fellow of the Population Council, the Guttmacher Institute, and the Royal College of Obstetricians and Gynaecologists. On a broader array of missions, he had been a member of several committees and panels with the National Academy of Sciences, such as the Committee on HIV Prevention Strategies in the United States, as the Panel on Data and Research Priorities for Arresting AIDS in Sub-Saharan Africa, the Committee on Antiprogestins, the Committee on National Statistics, the Committee on Population, and several Panels on Census, such as the Panel on Census Requirements in the Year 2000 and Beyond, Panel on the 1990 Census, Panel on Immigration Statistics, Panel on Small Area Estimation, and Panel on the 1980 Census. He also served for several years on the Council of the International Union for the Scientific Study of Population (1998–2005).

Professor Trussell spent his entire academic career at Princeton University, becoming a graduate student in 1973, assistant professor in 1975, and professor emeritus in 2015. In his final biosketch, he took care to highlight his valued role as teacher and mentor to many PhD students and postdoctoral trainees. He served many years as associate or acting dean of the Woodrow Wilson School and director of the Office of Population Research. He was an honorary fellow at the University of Edinburgh, Scotland, and a visiting professor at Hull York Medical School, England.

He was a Marshall scholar and a Watson Fellow. The short-list of his awards include a Scientific Contribution Award from the National Family Planning and Reproductive Health Association and a Carl S. Schultz Award from the American Public Health Association, the National Family Planning and Reproductive Health Association, the American Society of Emergency Contraception and International Consortium for Emergency Contraception.

Professor Trussell leaves behind a considerable legacy. His substantial volume of work will continue to inform our field for years to come (Table 1). He also leaves behind friends and colleagues who miss his drive and intensity (he was intense even in humor) and, most of all, his company.

**Table 1:** Key representative Landmark articles authored by James Trussell

*Contraceptive failure*	
Trussell J. (2011). Contraceptive failure in the United States. Contraception, May;83 (5):397–404. Epub 2011 Mar 12. PMCID: PMC3638209.	
Trussell J, Portman D. (2013). The creeping pearl: why has the rate of contraceptive failure increased in clinical trials of combined hormonal contraceptive pills? Contraception, Nov;88 (5):604–10. Epub 2013 Apr 11. PMCID: PMC3795840.	
Trussell J. (1991). Methodological pitfalls in the analysis of contraceptive failure. *Stat Med,* Feb;10 (2):201–20. PubMed PMID: 2052800.	
Trussell J, Vaughan B, Stanford J. (1999). Are all contraceptive failures unintended pregnancies? Evidence from the 1995 National Survey of Family Growth. *Fam Plann Perspect,* Sep-Oct;31 (5):246–7, 260. PubMed PMID: 10723650.	
*Emergency contraception*	
Trussell J, Stewart F, Guest F, Hatcher RA. (1992). Emergency contraceptive pills: a simple proposal to reduce unintended pregnancies. Fam Plann Perspect, Nov-Dec;24 (6):269–73. PubMed PMID: 1483531.	
Trussell J, Ellertson C, Dorflinger L. (2003). Effectiveness of the Yuzpe regimen of emergency contraception by cycle day of intercourse: implications for mechanism of action. Contraception, Mar;67 (3):167–71. PubMed PMID: 12618250.	
Trussell J, Raymond EG. (1999). Statistical evidence about the mechanism of action of the Yuzpe® regimen of emergency contraception. Obstet Gynecol, May;93 (5 Pt 2):872–6. PubMed PMID: 10912436.	
The Emergency Contraception Website (not-2-late.com) available at http://ec.princeton.edu/	
*Cost-effectiveness of contraception*	
Trussell J, Hassan F, Lowan J, Law A, Filonenko A. (2015). Achieving cost-neutrality with long-acting reversible contraceptive methods. Contraception, Jan;91 (1):49–56. Epub 2014 Sep 6. PMCID: PMC4268022.	
Trussell J. (2008). Overstating the cost savings from contraceptive use. Eur J Contracept Reprod Health Care, Sep;13 (3):219–21. PubMed PMID: 18821460.	
Trussell J, Lalla AM, Doan QV, Reyes E, Pinto L, Gricar J. (2008). Cost effectiveness of contraceptives in the United States. Contraception, Jan;79 (1):5–14. Epub 2008 Sep 25. PMCID:PMC3638200.	
Trussell J, Koenig J, Stewart F, Darroch JE. (1997). Medical care cost savings from adolescent contraceptive use. Fam Plann Perspect, Nov-Dec;29 (6):248–55, 295. PubMed PMID: 9429869.	
*Safety and efficacy of medical abortion*	
Fjerstad M, Trussell J, Sivin I, Lichtenberg ES, Cullins V. (2009). Rates of serious infection after changes in regimens for medical abortion. N Engl J Med., Jul 9;361 (2):145–51. PMCID:PMC3568698.	
Trussell J, Nucatola D, Fjerstad M, Lichtenberg ES. (2014). Reduction in infection-related mortality since modifications in the regimen of medical abortion. Contraception, Mar;89 (3):193–6. Epub 2013 Dec 11. PMCID: PMC3965643.	
Fjerstad M, Sivin I, Lichtenberg ES, Trussell J, Cleland K, Cullins V. (2009). Effectiveness of medical abortion with mifepristone and buccal misoprostol through 59 gestational days. Contraception, Sep;80 (3):282–6. Epub 2009 May 2. PMCID: PMC3766037.	
Fjerstad M, Trussell J, Lichtenberg ES, Sivin I, Cullins V. (2011). Severity of infection following the introduction of new infection control measures for medical abortion. Contraception, Apr;83 (4):330–5. Epub 2010 Oct 8. PMCID: PMC3758664.	
Aiken ARA, Gomperts R, Digol I, Trussell J. Self-Reported outcomes and adverse events following medical abortion via online telemedicine: a population-based study in Ireland and Northern Ireland. BMJ. 2017 May 16;357. PMCID: PMC5431774.	
*Estimating demographic parameters from inaccurate/incomplete data*	
Hill K, Zlotnik H, Trussell J. (1983). Manual X: Indirect Techniques for Demographic Estimation. Popul Stud, No. 81. New York NY: United Nations, Department of International, Economic, and Social Affairs; 1983. p. 304.	
Coale A, Trussell J. (1996). The development and use of demographic models. Popul Stud (Camb). Nov;50 (3):469–84. PubMed PMID: 11618377.	
Coale AJ, Trussell TJ. (1974). Model fertility schedules: Variations in the age structure of childbearing in human populations. Popul Index, Apr;40 (2):185–258.	
Coale AJ, Trussell J. (1977). Annex I: estimating the time to which Brass estimates apply. Popul Bull UN, (10):87–9. PubMed PMID: 12310593.	

## Data Availability

Not applicable.
